# Prediction of cervical intraepithelial neoplasia grade 2+ (CIN2+) using HPV DNA testing after a diagnosis of atypical squamous cell of undetermined significance (ASC-US) in Catalonia, Spain

**DOI:** 10.1186/1471-2334-12-25

**Published:** 2012-01-26

**Authors:** Raquel Ibáñez, Judit Moreno-Crespi, Montserrat Sardà, Josefina Autonell, Montserrat Fibla, Cristina Gutiérrez, Belen Lloveras, María Alejo, Isabel Català, Francesc Alameda, Miquel Casas, F Xavier Bosch, Silvia de Sanjosé

**Affiliations:** 1 Unit of Infections and Cancer. Cancer Epidemiology Research Programme, IDIBELL. Catalan Institute of Oncology, Av. Gran Vía, 199-203L'Hospitalet de Llobregat, 08907 Barcelona, Spain; 2 Pathology Department, University Hospital of Girona Dr. Josep Trueta, Catalan Institute of Oncology, Girona, Spain; 3 Pathology Department, Hospital Consortium of Vic, Barcelona, Spain; 4 Pathology Department, University Hospital of Tarragona Joan XXIII, Tarragona, Spain; 5 Clinical Laboratory ICS Tarragona, Molecular Biology Section University Hospital of Tarragona Joan XXIII IISPV, Rovira i Virgili University, Tarragona, Spain; 6 Pathology Department, Hospital del Mar, Barcelona, Spain; 7 Pathology Department, L'Hospitalet General Hospital, Barcelona, Spain; 8 Pathology Department, Bellvitge University Hospital, Barcelona, Spain; 9 CIBER Epidemiologia y Salud Pública, Madrid, Spain; 10 Universitat de Barcelona, Barcelona, Spain

**Keywords:** Human papillomavirus (HPV), Diagnosis of atypical squamous cell of undetermined significance (ASC-US), Triage, cervical cancer screening, hrHC2 testing

## Abstract

**Background:**

A protocol for cervical cancer screening among sexually active women 25 to 65 years of age was introduced in 2006 in Catalonia, Spain to increase coverage and to recommend a 3-year-interval between screening cytology. In addition, Human Papillomavirus (HPV) was offered as a triage test for women with a diagnosis of atypical squamous cells of undetermined significance (ASC-US). HPV testing was recommended within 3 months of ASC-US diagnosis. According to protocol, HPV negative women were referred to regular screening including a cytological exam every 3 years while HPV positive women were referred to colposcopy and closer follow-up. We evaluated the implementation of the protocol and the prediction of HPV testing as a triage tool for cervical intraepithelial lesions grade two or worse (CIN2+) in women with a cytological diagnosis of ASC-US.

**Methods:**

During 2007-08 a total of 611 women from five reference laboratories in Catalonia with a novel diagnosis of ASC-US were referred for high risk HPV (hrHPV) triage using high risk Hybrid Capture version 2. Using routine record linkage data, women were followed for 3 years to evaluate hrHPV testing efficacy for predicting CIN2+ cases. Logistic regression analysis was used to estimate the odds ratio for CIN2 +.

**Results:**

Among the 611 women diagnosed with ASC-US, 493 (80.7%) had at least one follow-up visit during the study period. hrHPV was detected in 48.3% of the women at study entry (mean age 35.2 years). hrHPV positivity decreased with increasing age from 72.6% among women younger than 25 years to 31.6% in women older than 54 years (*p *< 0.01).

At the end of the 3 years follow-up period, 37 women with a diagnosis of CIN2+ (18 CIN2, 16 CIN3, 2 cancers, and 1 with high squamous intraepithelial lesions -HSIL) were identified and all but one had a hrHPV positive test at study entry. Sensitivity to detect CIN2+ of hrHPV was 97.2% (95%confidence interval (CI) = 85.5-99.9) and specificity was 68.3% (95%CI = 63.1-73.2). The odds ratio for CIN2+ was 45.3 (95% CI: 6.2-333.0), when among ASC-US hrHPV positive women were compared to ASC-US hrHPV negative women.

**Conclusions:**

Triage of ASC-US with hrHPV testing showed a high sensitivity for the detection of CIN2+ and a high negative predictive value after 3 years of follow-up. The results of this study are in line with the current guidelines for triage of women with ASC-US in the target age range of 25-65. Non adherence to guidelines will lead to unnecessary medical interventions. Further investigation is needed to improve specificity of ASC-US triage.

## Background

Invasive cervical cancer (ICC) is the third most common tumor in women worldwide. Persistent infection with high risk Human Papillomavirus types (hrHPV) is considered a necessary cause for the development of ICC [1,2].

Cytology based cervical cancer (CC) screening has been fundamental in decreasing the incidence and mortality of cervical cancer in those countries with high screening coverage rates [3]. One of the most common abnormal diagnoses identified in cytology based cervical screening of asymptomatic women is atypical squamous cells of undetermined significance (ASC-US). The percentage of cytological results reporting the presence of ASC-US ranges from 5% in the USA to 2% in Sweden [3-6]. The management of ASC-US generally includes referral to colposcopy, with its subsequent cost and patient anxiety. Colposcopy examination should therefore be performed only in cases of clear clinical benefits. Data are now consistent in showing that triage with hrHPV DNA using Hybrid Capture 2 (HC2, Qiagen, Baltimore, USA) results in a higher sensitivity with a small decrease in specificity for cervical intraepithelial lesions grade 3 (CIN3) when compared to repeated cytology [3,5,7-10]. Most clinical algorithms for the triage of ASC-US triage now involve HPV testing [8-10].

Catalonia is a region in the North-East part of Spain with a population of 2,802,504 million women aged 25 and above in 2008. The incidence of ICC in 2008 was 7 per 100,000 representing 2.7% of all cancer cases in the region [11]. As in other regions of Spain, cervical cytology is recommended as the primary screening preventive strategy for ICC. This is offered under an opportunistic frame within the public sector. The coverage of screening cytology is estimated to be around 50-70%[12-14].

In 2006, HPV DNA testing for high risk types using Hybrid Capture version 2 (hrHC2) was introduced on a regular basis for the triage of ASC-US. The main aim of the present study was to evaluate, for the first time in Spain, the value of hrHPV as a predictor marker of CIN2+ lesions.

## Methods

Public health in Catalonia is free of direct charge for the population and provides full access to medical care and cancer screening interventions. About 70% of the gynaecological care in the population of Catalonia is carried out within the Public Sector.

In Catalonia, from June 2006 to October 2007 a new screening protocol for CC in sexually active women aged 25-65 years was implemented to increase coverage and to reach a 3 years interval between screening cytologies [14]. Younger women could also benefit of ICC screening after 3 years of sexual initiation. Further, women became eligible for hrHPV testing under the following circumstances: i) having an incident diagnosis of ASC-US; ii) being aged 40 years or more and having a poor cervical cancer screening history with no Pap smear in the previous 5 years, and iii) first follow up visit after a surgical conization. In the present study we reviewed the intervention among ASC-US cases. Evaluation of ii and iii is ongoing.

Primary care doctors and midwives were regularly required to evaluate the adequate compliance of women to the general screening recommendations detailed in the protocol.

As per protocol, follow-up of women with a screening cytological result of ASC-US in the absence of liquid-based cytology included recommendation for another cervical cells sample to evaluate the presence of high risk hrHPV within a 3 months period from the initial result. Follow-up after a 3 year interval was recommended for those women with negative hrHPV test results. For those women with positive hrHPV test result referral to colposcopy and biopsy (if appropriate) were recommended.

A total of 37,711 hrHPV tests were performed within 2007-2008 in the context of the population based screening protocol in Catalonia. Of these, 5,861 hrHPV tests were among women with a diagnosis of ASC-US. Among all participant centres, eight pre-defined geographical areas were selected for protocol monitoring: Baix Llobregat litoral, Osona, Bages, Berguedà, Solsonès, Sant Martí and Ciutat Vella districts of city of Barcelona, Gironès - Plà d'Estany and Tarragonès. Participating centers were consistent with the distribution of the urban/rural balance of the population in whole Catalonia.

HPV tests were performed in five referral laboratories. All of them followed an external quality control (QC) for the performance of hrHC2 testing. The Catalan Institute of Oncology was in charge of coordinating the QC. The QC analysis resulted in very high performance in all the laboratories (data not shown). The overall project was approved by the ethical committee of the Catalan Institute of Oncology.

### HPV DNA testing and pap smears

HPV DNA testing was conducted by five reference laboratories: Josep Trueta Hospital, Hospital Consortium of Vic, University Hospital of Tarragona Joan XXIII, Hospital del Mar, Bellvitge University Hospital. HPV detection was performed using hrHC2 tests (HC2, Qiagen, Baltimore, USA) which detect 13 high risk oncogenic HPV types (16, 18, 31, 33, 35, 39, 45, 51, 52, 56, 58, 59 and 68). An HPV sample was considered positive if it attained or exceeded the Federal Drug Administration (FDA)-approved threshold of 1.0 pg HPV DNA ml^-1^, which corresponds to 1.0 relative light unit (RLU/CO). Cervical cytologies were taken with conventional Pap smears or with liquid based cervical cytology and processed and read at the routine pathology centres. All the cytology results followed the 2001 Bethesda System [15] and they were classified as: 1) negative for intraepithelial lesion or malignancy, 2) ASC-US, atypical squamous cells cannot exclude a high grade squamous intraepithelial lesion (ASC-H), 3) low-grade squamous intraepithelial lesion (LSIL) encompassing human papillomavirus/mild dysplasia/cervical intraepithelial neoplasia (CIN) 1, 4) high-grade squamous intraepithelial lesion (HSIL) encompassing: moderate and severe dysplasia, carcinoma in situ, CIN 2 and CIN 3; 5) squamous cell carcinoma, atypical glandular cells (AGC) (specify endocervical, endometrial, or not otherwise specified), atypical glandular cells, favor neoplastic (specify endocervical or not otherwise specified), endocervical adenocarcinoma in situ (AIS) and adenocarcinoma.

For the histological diagnosis, the protocol guidelines recommended the use of CIN classification [16].

### Follow-up

All histological, cytological and molecular data of cases diagnosed with ASC-US were collected and evaluated to its final worse diagnosis using a record linkage approach based on the medical record number of the women. Once the information was available, all patient data were anonymised. Those women with negative hrHPV test after ASC-US diagnosis should be recommended a follow up visit in a 3 year interval. When a woman had any abnormal consecutive pathology test result after the initial ASC-US, the final diagnosis was the worst during the period and censored at the time of treatment, if any. In these later cases the assignment to a final diagnosis was based on the result of the biopsy before treatment. For those women with initial HPV positive test results, a final diagnosis of 'normal' was reported in patients with 1) two negative consecutive cytological samples, 2) two negative consecutive cervical biopsies, or 3) one negative cytological sample and one negative cervical biopsy within a 1 year interval or a second hrHPV negative test result. For those women with hrHPV negative results, a final diagnosis of "normal" was reported if at least one test result was negative at the end of follow up period. When both cytological and histological specimens were collected at the same time, the worst histological abnormality result was reported as final diagnosis.

### Statistical analysis

Data analyses were carried out using SPSS version 17.0. Age was grouped into five categories: < 25, 25-34, 35-44, and 45-54 and ≥ 54 years old. The relative light units/cut off (RLU/CO) was divided into nine categories: 0-0.5, 0.6-0.99, 1-1.99, 2-49, 50-99, 100-499, 500-999, 1000-1999, ≥ 2000. Sensitivity, specificity, positive predictive value (PPV), negative predictive value (NPV) were estimated together with exact 95% CI, one or two sided as appropriate. When appropriate, comparison between categories was performed with a *t*-test for continuous variables and a chi square test for categorical variables. Finally, a logistic regression analysis adjusted by age and centre was performed in order to calculate the odds ratio of developing a CIN2+ lesion as a function of hrHPV status at study entry. The analysis was performed as a cross-sectional evaluation at the end of the first round of screening.

## Results

During 2007 and 2008, 65,378 women from the study population were screened with conventional cytology and a total of 69,952 Pap smears were obtained. Out of them, 95% were negative for cervical intraepithelial lesion or malignancy, 2% were reported as ASC-US/atypical glandular cells of undetermined significance (AGUS), 0.08% were reported as atypical squamous cells-cannot exclude HSIL (ASC-H), 2% were reported as LSIL, 0.3% were reported as HSIL and 0.03% were suspected of cancer. 613 ASC-US cases that had a concomitant hrHPV testing were included in the present study (76 from the counties of Osona, Bages, Berguedà and Solsonès, 105 from the counties of Gironès and Pla de l'Estany, 213 from the counties of Baix Llobregat Litoral, 151 from the Barcelona districts of Sant Martí and Ciutat Vella and 68 from the county of Tarragonès). Two cases were excluded: one with history of vaginal cancer and the other with endometrial cancer. The final sample size for the analyses was of 611 women. All the hrHPV tests were performed within a maximum period of 6 months after ASC-US diagnosis. The 68 cases of Tarragonès were included despite of being diagnosed in 2008 because follow-up was available for the majority of the women (81%).

The mean age of ASC-US cases was 35.4 years (standard deviation (SD) = 11.3) ranging from 15 to 79 years (Table 1). Follow-up information was available for 493 women (80.7% of the total sample). There was no difference in the age distribution of women with and without follow-up information. Women with follow-up were more likely to have a hrHPV positive test result (48.3%) in comparison with ASC-US hrHPV negative women (51.7%, *p *= 0.02).

**Table 1:** General characteristics of women with a diagnosis of ASC-US from 5 sentinel screening centres in Catalonia (Spain)

CHARACTERISTIC	TOTAL	WOMEN WITH FOLLOW UP^&^
**Total cases**	611	493

**Mean follow up time years**	-	3.9

**Age mean years (range)**	35.4(15-79)	35.2(15-71)

**Age groups yrs**	**N(%)**	**N(%)**

< 25	106(17.3)	84(17.0)

25-34	224(36.7)	182(36.9)

35-44	125(20.4)	100(20.3)

45-54	126(20.6)	108(21.9)

> 54	30(4.9)	19(3.8)

**First hrHPV after ASC-US**	**N(%)**	**N(%)**

Negative	331(54.2)	255(51.7)

Positive	280(45.8)	238(48.3)*

^&^Maximum follow up period 3.5 years * *p *value hetereogeneity test 0.02

ASC-US: Atypical squamous cell of undetermined significance, hrHPV: High risk Human Papillomavirus test

Table 2 shows the final diagnosis at the end of the follow-up period by initial HPV DNA result. Among hrHPV positive cases, 45.4% had a final diagnosis of negative for CIN, 15.5% remained hrHPV positive with no other cytological abnormality, and 14.4% had a diagnosis of HSIL-CIN2+ (36 out of 238). One squamous carcinoma (stage 2) and one mucinous adenocarcinoma (stage 3) were also identified in this group. On the contrary, among HPV negative women, 91.4% had a final diagnosis of negative for CIN and one case (0.4%) was diagnosed as CIN2. Further verification of this case showed a p16^INK ^immunochemistry positive test result suggestive of an HPV driven lesion. No sample was available for further testing.

**Table 2:** Diagnosis at last follow up^&^among 493 women with ASC-US from 5 sentinel screening centres in Catalonia (Spain) by hrHPV status

	TOTAL CASES	POSITIVE hrHPV		NEGATIVE hrHPV	
**Diagnosis at last follow up**^**& 1**^	**N**	**N**	**%**	**N**	**%**

Normal	341	108	31.7	233	68.3

**No histology confirmation:**					

ASC-US	13	6	46.2	7	53.8

hrHPV+	46	37	80.4	9	19.6

CIN-NOS	1	1	100.0	0	0.0

LSIL	33	30	90.9	3	9.1

HSIL*	1	1	100.0	0	0.0

**With histology confirmation:**					

CIN1	22	20	90.9	2	9.1

CIN-2	18	17	94.4	1	5.6

CIN-3	16	16	100.0	0	0.0

Cancer	2	2	100.0	0	0.0

**Total**	493	238	48.3	255	51.7

^&^Maximum follow up 3.5 years

^1 ^ASC-US: Atypical squamous cell of undetermined significance, HPV+: positive for Human Papillomavirus test, CIN-NOS: CIN not otherwise specified, CIN1: high grade cervical intraepithelial lesions grade 1, LSIL: low grade squamous intraepithelial lesion, CIN1: high grade cervical intraepithelial lesions grade 1, CIN2: high grade cervical intraepithelial lesions grade 2, CIN3: high grade cervical intraepithelial lesions grade 3, HSIL: High grade squamous intraepithelial lesion

* The HSIL case could not be specified as CIN2 or CIN3

The sensitivity of hrHC2 for the detection of histologically confirmed CIN2+ was of 97.2% (CI = 85.5-99.9) (35/36); and of 100% (CI_one sided _= 82.9) for CIN3+ (16/16). The specificity of hrHC2 for the detection of histologically confirmed CIN2+ was of 68.3% (CI = 63.1-73.2) (108/341), excluding intermediate diagnoses such as HPV positivity or LSIL. The NPV of hrHPV test for CIN2+ was of 99.6% (CI_one sided _= 98.2) (254/255).

Table 3 shows the distribution of final diagnosis by age group. The percentage of hrHPV positive cases was highest among women younger than 25 years (61/84, 72.6%) and declined thereafter (p value for linear trend < 0.01). The two cancer cases were diagnosed in 51 years old woman and in a 55 years old woman. CIN3 and HSIL cases clustered largely in the age range of 25 to 44 years, while CIN2 cases were mainly observed among women younger than 34 years old. Among ASC-US-HPV negative, the only CIN2 case was diagnosed in a 35 years old woman.

**Table 3:** Distribution of cervical abnormalities at the end of follow-up^&^by age and hrHPV status at entry among 493 women with ASC-US from 5 sentinel screening centres in Catalonia (Spain)

	AGE				
**DIAGNOSIS AT LAST FOLLOW UP**^&^ **BY hrHPV STATUS**	**< 25 N** **(%)**	**25-34** **N (%)**	**35-44** **N (%)**	**45-54** **N (%)**	**> 54** **N (%)**

**Total** N (% HPV positive)*	84(17) 61(72.6)	182(36.9) 101(55.5)	100(20,3) 38(38.0)	108(21.9) 32(29.6)	19(3.9) 6(31.6)

**hrHPV Positive**	**N (%) within diagnostic category**				

Cancer (N = 2)	0	0	0	1(50.0)	1(50.0)

CIN3/HSIL (N = 17)	3(17.6)	7(41.1)	4(23.5)	3(17.6)	0(0)

CIN2 (N = 17)	5(29.4)	8(47.0)	2(11.8)	1(5.9)	1(5.9)

Other milder diagnosis (N = 94)	19(20.2)	46(48.9)	19(20.2)	9(9.6)	1(1.1)

Normal (N = 108)	34(31.5)	40(37.0)	13(12.0)	18(16.7)	3(2.8)

Total (N = 238)	61(25.6)	101(42.4)	38(15.9)	32(13.4)	6(2.5)

**hrHPV Negative**	**N (%) within diagnostic category**				

CIN 2+ (N = 1)	0	0	1 (100)	0	0

Other milder diagnosis (N = 35)	7(20.0)	10(28.6)	9(25.7)	9(25.7)	0

Normal (N = 220)	16(7.2)	71(32.3)	53(24.1)	67(30.4)	13(5.9)

Total (N = 255)	23(9.0)	81(31.8)	62(24.3)	76(29.8)	13(5.1)

^&^Maximum follow up period 3.5 years

• *P *value for linear trend < 0.0

CIN2: high grade cervical intraepithelial lesions grade 2, CIN3: high grade cervical intraepithelial lesions grade 3, HSIL: High grade squamous intraepithelial lesion

Among hrHPV positive women with a final diagnosis of CIN2+, 69.4% were diagnosed as CIN2+ at first biopsy. The remaining cases were diagnosed within the established 6-12 months follow-up period for women with ASC-US results and being hrHPV positive and colposcopy negative or having a negative biopsy.

hrHPV status at study entry was the only statistical significant variable associated to the risk of CIN2+ (*p*-value < 0.05). The odds ratio of CIN2+ was 45.3 (95% CI = 6.2-333.0) when ASC-US hrHPV positive women were compared to that of ASC-US hrHPV negative women.

Among hrHPV positive cases, the lag time from ASC-US to CIN2+ was shorter and statistically significant in comparison with the lag time between ASC-US and other milder diagnostics (11.9 months versus 17.9 months, respectively, *p *value = 0.002).

Among the 255 ASC-US hrHPV negative women, 85.1% were at least examined once by a gynaecologist and a cervical smear was taken within the 3 year recommended lag period. The record linkage system identified 321 Pap smears, 34 histological specimens and 56 hrHPV tests performed during that period. The average lag time from the initial ASC-US HPV negative result to the next gynaecological test was 1.9 years (SD = 0.94).

## Discussion

The results of this study confirm that among women with a new diagnosis of ASC-US, hrHPV DNA testing clearly defines the group of women with an underlying high risk of CIN2+ with a PPV of 14.3% and a global NPV of 99.6% after a 3 years follow up period. hrHPV testing among women with ASC-US identified a high number of CIN2+ (37/493) cases conferring a risk of CIN2+ 45 times higher than the one observed in ASC-US negative women. Sensitivity for CIN2+ was of 97.3% and sensitivity for CIN3+ was of 100%. Specificity for CIN2+ was of 68.3%.

ASC-US is generally described to be more common in young women than in postmenopausal women [17,18]. In this study, 17% of the ASC-US cases were diagnosed in women younger than 25 years old. In this age group HPV was detected in a much larger proportion than in other age groups (72.6%), suggesting that acute infections are probably playing a major contribution in this age group. However, the proportion of CIN3+ was similar among women younger than 25 years old if compared to the other age groups (3.6% versus 3.9%, respectively). We cannot exclude that CIN3 lesions in this younger age group are more likely to regress spontaneously. Any woman with an ASC-US and hrHPV positive test is referred to colposcopy and close follow-up, while the likelihood of developing an ICC is extremely low in women younger than 25 years old. According to our protocol guidelines ASC-US, hrHPV positive women with a negative colposcopy will still need to be visited at 6 and 12 months and be retested for hrHPV at 12 months. Moscicki et al. [19], in a prospective study of CIN2 in adolescents and young women (< 25 years old), showed that frequently CIN2 regressed spontaneously supporting the conservative approach for this age group.

The majority of women with hrHPV negative values (91.4%) did not develop any pathology at the end of the first round of screening. According to previous studies [8,20,21], hrHPV negative women are very unlikely to develop a CIN2+ and it has been suggested that screening intervals would be safe if they are lengthened at least up to 6 years. Our findings show that a 3 year screening interval is safe although based on a small sample of women. hrHPV triage is likely to result in a cost-effective strategy if screening intervals are kept and diverts women from colposcopy in hrHPV negative women [10,22]. However, although the sensitivity of hrHC2 was very high, specificity was relatively low increasing unnecessarily the number of women referred to colposcopy because of a positive test. There was no indication that levels of RLU/CO could improve our specificity in the triage of ASC-US cases. It is likely that in the near future new triage strategies will improve specificity while keeping high sensitivity rates [21]. Further, in our study, 65.6% (217/331) of the total women with ASC-US hrHPV negative test had at least one follow-up visit before finishing the first round of screening, meaning that the current screening protocol recommending follow-up after a 3 years period is not widely subscribed by the professionals. In view of our data we can conclude that a bulk of additional tests were unnecessary, thus reducing the potential cost-effectiveness of the strategy. In addition unnecessary testing may induce anxiety, worry and discomfort in these patients.

Our data are consistent with other studies using hrHC2 as a triage test [23-26]. Del Mistro et al. [23] carried out a study in Veneto region (Italy) among 749 women with ASC-US cytology in an organized screening programme. The prevalence of hrHPV infection was of 42.9% and the mean age of the women in this study was of 42 years old. CIN2+ developed in 14.9% of the hrHPV positive women at enrolment and in only 0.35% of the hrHPV negative women. In our study, with slightly younger women and similar hrHPV prevalence, 15.1% of the hrHPV positive women developed a CIN2+ at the end of the follow up period and only 0.4% of the hrHPV negative women developed a CIN2+. In the ASCUS and LSIL triage study (ALTS) [5] which included 1161 ASC-US cases, 50.7% were hrHPV positive and 16.0% of them developed a CIN2+. Only 1.4% of the hrHPV negative women developed a CIN3 after a follow-up period of 2 years. Furthermore, in a study from an organized screening programme in the county of Gävle (Sweden) [6], hrHPV prevalence among ASC-US cases was of 50%, and 32.3% of the hrHPV positive cases and 2.04% of the HPV negative cases had a CIN2/3. The study results published in the scientific literature are consistent in observing that CIN2+ cases are almost universally within the hrHC2 positive category.

The limitations of the present study are that the follow-up window was slightly over a 3 years period - from January 2007 up to February 2011. Although information from 80.7% of the women was available, it is possible that appointments for the remaining ones were fixed later than the 3 years period. An additional limitation is that colposcopies among hrHPV negative women were not randomly carried out. However, a relevant proportion of these patients obtained negative colposcopy results for CIN2+ suggesting that verification bias was not affecting our results. Our data was restricted to information generated within the public health system serving over 70% of the gynaecological care. Therefore, we cannot extrapolate our data to women being attended in the private sector as they may differ in terms of risk factors from HPV acquisition or cervical cancer.

In this study we used the information from five sentinel centres with available record linkage system and no detailed medical records were available. We did not review the pathology diagnosis of the initial cytology of ASC-US nor the follow-up diagnosis by cytology, biopsy or hrHPV testing. Therefore, discrepancies between pathologists in classifying ASC-US cannot be excluded.

A strength of our study was that information was retrieved from a public health system in which a common protocol was introduced and monitored for its adequacy and performance across the different laboratories and medical centers. The protocol was homogenously introduced and monitored and coordinated irrespective of the clinical decisions taken.

## Conclusions

HPV DNA testing as a triage test for ASC-US has been shown to predict CIN2+ lesions and to provide very high negative predictive value among those cases that followed the protocol guidelines. This study supports our current national recommendations for ASC-US triage in women older than 25 years of age. Guidelines for younger women entering into screening protocols need to be re-evaluated. Non adherence to guidelines will lead to unnecessary medical interventions and psycho-social effects, such as anxiety. Further research is needed to improve specificity of ASC-US triage.

## Abbreviations

CC: Cervical cancer; HPV: Human Papillomavirus; hrHPV: High risk HPV types; ASC-US: Atypical squamous cells of undetermined significance; HC2: Hybrid Capture System; hrHC2: Hybrid Capture version 2 for high risk types detection; RLU/CO: Relative light unit; CIN2+: High grade cervical intraepithelial lesions grade 2 or worse; ASC-H: Atypical squamous cells cannot exclude a high grade squamous intraepithelial lesion; LSIL: Low-grade squamous intraepithelial; HSIL: High-grade squamous intraepithelial lesion; CIN2: High grade cervical intraepithelial lesions grade 2; CIN3: High grade cervical intraepithelial lesions grade 3; AGC: Atypical glandular cells; AIS: Endocervical adenocarcinoma in situ.

## Competing interests

Silvia de Sanjose received occasional travel fund to conferences/symposia/meetings by either GlaxoSmithKline, Sanofi Pasteur MSD, Merck & Co. or Qiagen.

F. Xavier Bosch is member of the advisory board of GlaxoSmithKline, Merck Sharp & Dohme, and Sanofi Pasteur MSD and of the speakers bureau of GlaxoSmithKline. He received occasional travel fund to conferences/symposia/meetings by either GlaxoSmithKline, Sanofi Pasteur MSD, Merck & Co. or Qiagen.

Belen Lloveras has received occasional travel fund to conferences/symposia/meetings are occasionally granted by either Qiagen or Roche

All other authors refer no conflict of interest.

## Authors' contributions

RI and SDS drafted the manuscript and performed the statistical analysis. All authors contributed to the collection and quality of data and read and approved the final manuscript.

## Pre-publication history

The pre-publication history for this paper can be accessed here:

http://www.biomedcentral.com/1471-2334/12/25/prepub

## References

[B1] Walboomers JM Jacobs MV Manos MM Bosch FX Kummer JA Shah KV Snijders PJF Peto J Meijer CJLM Muñoz N Human papillomavirus is a necessary cause of invasive cervical cancer worldwide J Pathol 1999 189 12 19 10.1002/(SICI)1096-9896(199909)189:1<12::AID-PATH431>3.0.CO;2-F 10451482

[B2] Ferlay J Parkin DM Curado MP International Agency for Research on Cancer Cancer Incidence in Five Continents 2010 I to IX (Accessed April, 2011 http://ci5.iarc.fr)

[B3] IARC Working Group on the Evaluation of Cancer-Preventive Strategies IARC Press Cervix Cancer Screening 2005 10 Lyon, France

[B4] Dufresne S Sauthier P Mayrand MH Petignat P Provencher D Drouin P Gauthier P Dupuis MJ Michon B Ouellet S Hadjeres R Ferenczy A Franco EL Coutlée F Human Papillomavirus (HPV) DNA Triage of Women with Atypical Squamous Cells of Undetermined Significance with Amplicor HPV and Hybrid Capture 2 Assays for Detection of High-Grade Lesions of the Uterine Cervix J Clin Microbiol 2011 49 1 48 53 10.1128/JCM.01063-10 21084508 PMC3020412

[B5] ASCUS-LSIL Triage Study (ALTS) Group Results of a randomized trial on the management of cytology interpretations of atypical squamous cells of undetermined significance Am J Obstet Gynecol 2003 188 6 1383 1392 12824967 10.1067/mob.2003.457

[B6] Silverloo I Andrae B Wilander E Value of high-risk HPV-DNA testing in the triage of ASCUS Acta Obstet Gynecol Scand 2009 88 9 1006 1010 10.1080/00016340903160952 19690991

[B7] Arbyn M Sasieni P Meijer CJ Clavel C Koliopoulos G Dillner J Clinical applications of HPV testing: a summary of meta-analyses Vaccine 2006 24 Suppl 3 S3/78 89 16950021 10.1016/j.vaccine.2006.05.117

[B8] Dillner J Rebolj M Birembaut P Petry KU Szarewski A Munk C de Sanjose S Naucler P Lloveras B Kjaer S Cuzick J van Ballegooijen M Clavel C Iftner T Joint European Cohort Study Long term predictive values of cytology and human papillomavirus testing in cervical cancer screening: joint European cohort study BMJ 2008 13 337 18852164 10.1136/bmj.a1754 PMC2658827

[B9] Cox JT History of the use of HPV testing in cervical screening and in the management of abnormal cervical screening results J Clin Virol 2009 45 Suppl 1 S3 S12 19651367 10.1016/S1386-6532(09)70002-2

[B10] Ostensson E Fröberg M Hjerpe A Zethraeus N Andersson S Economic analysis of human papillomavirus triage, repeat cytology, and immediate colposcopy in management of women with minor cytological abnormalities in Sweden Acta Obstet Gynecol Scand 2010 89 10 1316 1325 10.3109/00016349.2010.512066 20846064

[B11] Marcos-Gragera R Cardó X Galceran J Ribes J Izquierdo A Borràs J Cancer incidence in Catalonia, 1998-2002 Med Clin (Barc) 2008 131 Suppl 1 4 10 19080809 10.1016/s0025-7753(08)76427-3

[B12] Ascunce N Salas D Zubizarreta R Almazán R Ibáñez J Ederra M representatives of the Network of Spanish Cancer Screening Programmes (Red de Programas Españoles de Cribado de Cáncer) Cancer screening in Spain Annals of Oncology 2010 21 Supplement 3 iii43 iii51 10.1093/annonc/mdq085 20427360

[B13] Puig-Tintoré LM Castellsagué X Torné A de Sanjosé S Cortés J Roura E Méndez C Bosch FX Coverage and factors associated with cervical cancer screening: results from the AFRODITA study: a population-based survey in Spain J Low Genit Tract Dis 2008 12 2 82 89 10.1097/LGT.0b013e3181599c16 18369300

[B14] Departament de Salut Protocol de les Activitats per al Cribratge del Càncer de Coll Uterí a l'Atenció Primària Barcelona: Direcció General de Planificació i Avaluació 2006 Generalitat de Catalunya:Barcelona, Spain http://www20.gencat.cat/docs/canalsalut/Home%20Canal%20Salut/Professionals/Recursos/Protocols_i_recomanacions/27_cancer/documents/Protocol_activitats_cribratge_%20cancercolluteri_atencioprimaria.pdf

[B15] Solomon D Davey D Kurman R Moriarty A O'Connor D Prey M Raab S Sherman M Wilbur D Wright T Jr Young N Forum Group Members Bethesda 2001 Workshop. The 2001 Bethesda System: terminology for reporting results of cervical cytology JAMA 2002 287 16 2114 2119 10.1001/jama.287.16.2114 11966386

[B16] Bulten J Horvat R Jordan J Herbert A Wiener H Arbyn M European guidelines for quality assurance in cervical histopathology Acta Oncol 2011 50 5 611 620 10.3109/0284186X.2011.555779 21314297

[B17] Wright TC Jr Massad LS Dunton CJ Spitzer M Wilkinson EJ Solomon D 2006 ASCCP-Sponsored Consensus Conference: 2006 consensus guidelines for the management of women with abnormal cervical screening tests J Low Genit Tract Dis 2007 11 4 201 222 10.1097/LGT.0b013e3181585870 17917566

[B18] Apgar BS Kittendorf AL Bettcher CM Wong J Kaufman AJ Update on ASCCP consensus guidelines for abnormal cervical screening tests and cervical histology Am Fam Physician 2009 80 2 147 155 19621855

[B19] Moscicki AB Ma Y Wibbelsman C Darragh TM Powers A Farhat S Shiboski S Rate of and risks for regression of cervical intraepithelial neoplasia 2 in adolescents and young women Obstet Gynecol 2010 116 6 1373 1380 10.1097/AOG.0b013e3181fe777f 21099605 PMC3057366

[B20] Meijer CJ Berkhof H Heideman DA Hesselink AT Snijders PJ Validation of high-risk HPV tests for primary cervical screening J Clin Virol 2009 46 Suppl 3 S1 S4 20129067 10.1016/S1386-6532(09)00540-X

[B21] Cuzick J Arbyn M Sankaranarayanan R Tsu V Ronco G Mayrand MH Dillner J Meijer CJ Overview of human papillomavirus-based and other novel options for cervical cancer screening in developed and developing countries Vaccine 2008 26 Suppl 10 K29 K41 18847555 10.1016/j.vaccine.2008.06.019

[B22] Diaz M de Sanjose S Ortendahl J O'Shea M Goldie SJ Bosch FX Kim JJ Cost-effectiveness of human papillomavirus vaccination and screening in Spain Eur J Cancer 2010 46 16 2973 2985 10.1016/j.ejca.2010.06.016 20638840

[B23] Del Mistro A Frayle-Salamanca H Trevisan R Matteucci M Pinarello A Zambenedetti P Buoso R Fantin GP Zorzi M Minucci D Triage of women with atypical squamous cells of undetermined significance (ASC-US): results of an Italian multicentric study Gynecol Oncol 2010 117 1 77 81 10.1016/j.ygyno.2010.01.003 20116836

[B24] Bhatla N Moda N The clinical utility of HPV DNA testing in cervical cancer screening strategies Indian J Med Res 2009 130 3 261 265 19901435

[B25] Luyten A Scherbring S Reinecke-Lüthge A Braun BE Pietralla M Theiler K Petry KU Risk-adapted primary HPV cervical cancer screening project in Wolfsburg, Germany-experience over 3 years J Clin Virol 2009 46 Suppl 3 S5 S10 20129072 10.1016/S1386-6532(09)70294-X

[B26] Mesher D Szarewski A Cadman L Cubie H Kitchener H Luesley D Menon U Hulman G Desai M Ho L Terry G Williams A Sasieni P Cuzick J Long-term follow-up of cervical disease in women screened by cytology and HPV testing: results from the HART study Br J Cancer 2010 102 9 1405 1410 10.1038/sj.bjc.6605619 20354519 PMC2865748

